# Trends in the Prevalence of Morbid and Severe Obesity in Australian Children Aged 7-15 Years, 1985-2012

**DOI:** 10.1371/journal.pone.0154879

**Published:** 2016-05-12

**Authors:** Sarah P. Garnett, Louise A. Baur, Aimee M. D. Jones, Louise L. Hardy

**Affiliations:** 1 Institute of Endocrinology and Diabetes and Kids Research Institute at the Children's Hospital at Westmead, Sydney, Australia; 2 The Children's Hospital at Westmead Clinical School, University of Sydney, Sydney, NSW, Australia; 3 Prevention Research Collaboration, School of Public Health, University of Sydney, Sydney, NSW, Australia; Swinburne University of Technology, AUSTRALIA

## Abstract

**Objective:**

Children with severe obesity have greater risk of adverse health outcomes. The purpose of this study was to assess trends in the prevalence of morbid and severe obesity in Australian children between 1985 and 2012.

**Methods:**

Secondary analysis of four national Australian cross-sectional surveys of measured height/weight in 7–15 year olds: Australian Health and Fitness Survey 1985 (n = 8,486), National Nutrition Survey 1995 (n = 1,541), the National Children’s Nutrition and Physical Activity Survey 2007 (n = 2,585) and the National Health Survey 2012 (n = 2,940). International Obesity Taskforce cut-point was used for morbid obesity (equivalent to a BMI ≥35kg/m^2^ at age 18 years). Severe obesity class 2 was defined as BMI ≥120% and <140% of the 95th percentile of the CDC 2000 growth charts or a BMI ≥35 and <40, and severe obesity class 3 as BMI ≥140% of the 95th percentile or a BMI ≥40.

**Results:**

Between 1985 and 2012 the prevalence of morbid obesity increased from 0.2% to 1.8%, class 2 severe obesity from 0.3% to 2.0%, and class 3 from 0.1% to 0.5%. Children with morbid obesity represented 11.3% of children with obesity in 1985 and increased to 22.5% in 2012 (P = 0.005). Children with severe obesity represented 19.3% of children with obesity in 1985 and increased to 32.0% in 2012 (P = 0.016). The greatest increase was observed between 1995 and 2007. The proportion of children who were classified as morbidly or severely obese was not significantly different between 2007 and 2012, nor was it significantly different between age and sex groups.

**Conclusion:**

Prevalence of morbid and severe obesity among children is low, but has significantly increased between 1985 and 2012. In contrast to overweight and obese children, children with morbid obesity require tertiary intervention. Failure to treat these children will have significant implications for the individual child and community.

## Introduction

Emerging evidence suggests that the increasing rates of obesity in children and adolescents, as measured by body mass index (BMI), may be slowing or even plateauing in several countries, including Australia and the US [1]. However, there is concern that the BMI distribution curve has shifted further to the right, indicating that the proportion of children with morbid obesity (equivalent to age and sex adjusted BMI ≥35 kg/m2 at 18 years [2] and severe obesity (BMI ≥ 120% of the 95th percentile or a BMI ≥35 kg/m2 of CDC 2000 growth charts) is increasing [3]. In the US, severe obesity is one of the fastest growing subcategories of obesity in children age 2–19 years, with rates of severe obesity increasing from 4.7% to 8.0% between 1999/2000 and 2011/2012 [4]. Children with severe obesity have a greater risk of serious short and long term cardiovascular, metabolic and other health consequences compared with children and who are overweight or obese [3].

Monitoring trends in the severity of obesity is essential to predict the associated disease burden and inform options for intervention. The prevalence rates of morbid and severe obesity in Australian children have not been reported previously. The aim of this study was to report changes in morbid and severe obesity in Australian children age 7 to 15 years between 1985 and 2012.

## Methods

Secondary data analysis of measured height and weight data of children age 7 to15 years from four Australian representative cross-sectional surveys: the Australian Health and Fitness Survey 1985 (AHF85) [5], the National Nutrition Survey 1995 (NNS95) [6], the 2007 Australian National Children’s Nutrition and Physical Activity Survey (NCNPAS07) [7] and the Australian National Health Survey 2012 (NHS12) [8]. Details of AHF85, NNS95 and NCNPA07 have been described previously in detail [9]. Briefly, the AHF85 was a national population survey with measured height and weight of 8,486 school children aged 7 to 15years (response rate, 67.5%). The NNS95 was a joint project of the Australian Bureau of Statistics and the then Commonwealth Department of Health and Aged Care, with measured height and weight on 1,541 children aged 7 to 15years (response rate, 61%). The NCNPAS07 (n = ~4,490) was commissioned by the Department of Health and Ageing, the Department of Agriculture and Fisheries and Forestry, and the Australian Food and Grocery Council and included measured height and weight of 2,585 children aged 7 to 15years (response rate, 40%). The NHS12 was conducted by the Australian Bureau of Statistics. The overall response rate was 85% of which 83% had weight and height measured. Data on young people aged 7 to 15 years were included in this analysis (n = 2,940) and were provided by the Australian Bureau of Statistics as a confidentialised unit record file.

Ethics approval for the studies was obtained from the State Directors General of Education (AHF85), the Australian Institute of Health and Welfare (NNS95) or the Australian Commonwealth Scientific Research Organization and the University of South Australia (NCNPAS07) and the Department of Health and Aging (NHS12).

### Definition of morbid and severe obesity

There is no consensus on the definition of severity of obesity, so we applied the most recent International Obesity Taskforce (IOTF) BMI cut-points for morbid obesity (equivalent to age and sex adjusted BMI≥ 35kg/m^2^ at 18 years) [2] and the American Heart Association definition for severe obesity class 2, defined as BMI ≥120% and <140% of the 95th percentile of the CDC 2000 growth references, or a BMI ≥35kg/m^2^, whichever was lower, and severe obesity class 3,defined as BMI ≥140% of the 95th percentile or a BMI ≥ 40 kg/m^2^, whichever was lower [3].

### Age groups

To allow comparison with previous Australian studies [9,10] data were categorised by age groups, 7 to 11 years and 12 to 15 years.

### Statistical analysis

Data were analysed using the PAWS Statistical Package version 21.0.0 (http://www-01.ibm.com/software/analytics/spss). Differences in categorical data were assessed by Chi squared tests. The effects of age and sex were examined by logistic regression.

## Results

**Fig 1:**
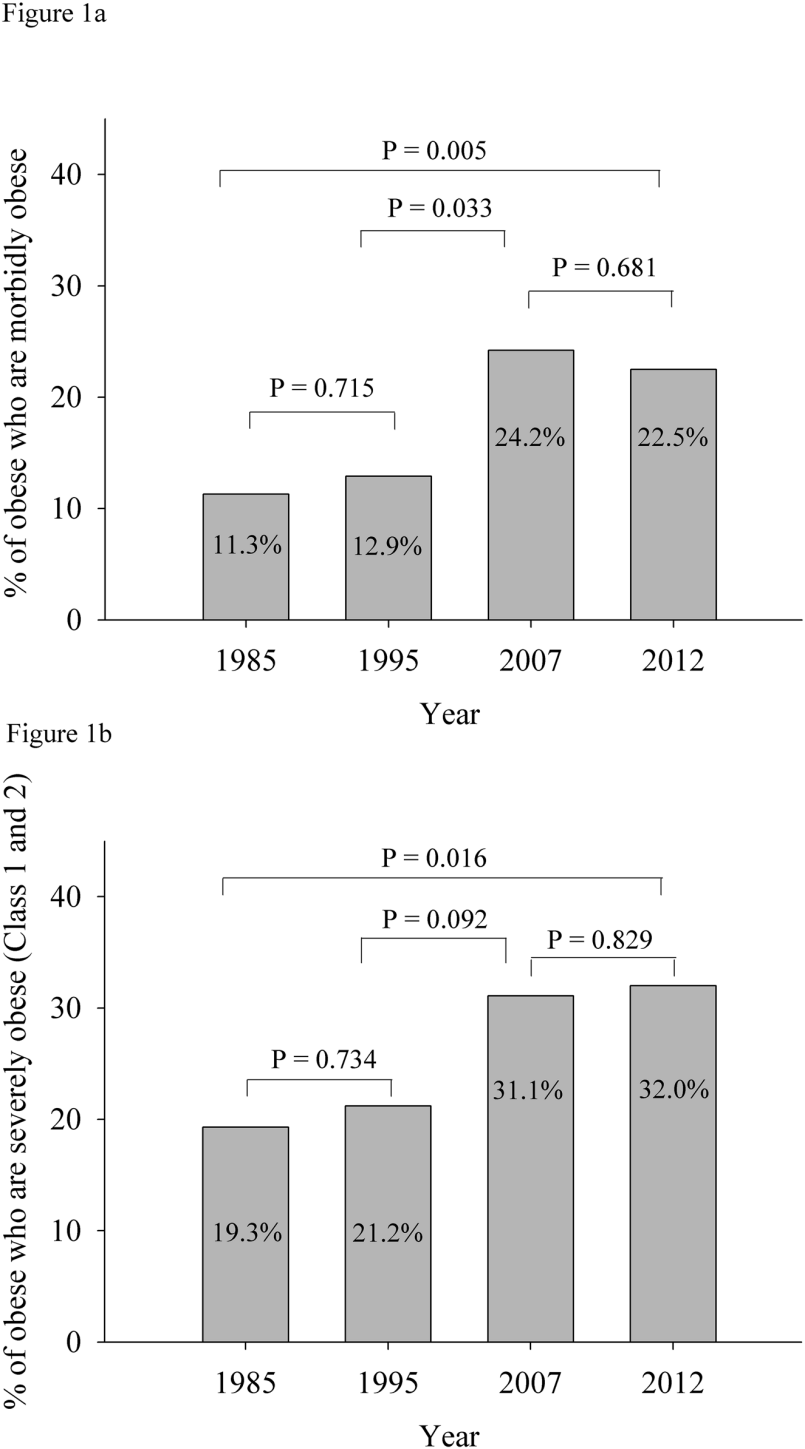
Prevalence of morbid obesity (Fig 1a) and prevalence of severe obesity (Fig 1b) among children with obesity 1985, 1995, 2007 and 2012. Morbid obesity was defined as age and sex adjusted BMI≥ 35kg/m^2^ at 18 years) (2) and severe obesity included class 2 and 3 and was defined as BMI ≥120% 95th percentile of the CDC 2000 growth references, or a BMI ≥35kg/m^2^, whichever was lower (3).Statistical significance was determined by chi-squared test.

Between 1985 and 2012 the prevalence of obesity in children increased over two and a half fold, the prevalence of morbid obesity eightfold, and severe obesity fivefold (class 2) and fourfold (class 3), Table 1. The proportion of obese children who were classified as morbidly or severely obese did not change significantly between 1985 and 1995 nor did the proportion change significantly between 2007 and 2012, Fig 1a and 1b. However, the proportion of obese children who were morbidly obese significantly (P = 0.033) increased from 12.9% in 1995 to 24.2% in 2007, Fig 1a. Similarly, during this time, severe obesity (both class 2 and 3) increased from 21.1% to 31.1% (P = 0.092) of obese children, Fig 1b. The proportion of children who were classified as obese, or morbidly or severely obese was not significantly different between age and sex groups.

**Table 1:** Characteristics and BMI status of Australian children age 7 to 15 years 1985, 1995, 2007 and 2012.

	AHF85	NNS95	NCNPAS07	NHS12
	n (%)	n (%)	n (%)	n (%)
**Sex**				
Boys	4301 (50.7)	815 (52.9)	1275 (49.3)	1480 (50.3)
Girls	4185 (49.3)	726 (47.1)	1310 (50.7)	1460 (49.7)
Total	8486	1541	2585	2940
**Age (years)—Boys**				
7 to 11	2419 (28.5)	457 (29.6)	603 (23.4)	812 (27.6)
12 to 15	1882 (22.2)	358 (23.3)	672 (25.9)	668 (22.7)
**Age (years)—Girls**				
7 to 11	2439 (28.7)	438 (28.4)	634 (24.6)	770 (26.2)
12 to 15	1746 (20.6)	288 (18.7)	676 (26.1)	690 (23.5)
**IOTF BMI category**				
Healthy weight^a^	7472 (88.1)	1200 (77.9)	1927 (74.6)	2142 (72.9)
Overweight^b^	864 (10.2)	256 (16.6)	468 (18.1)	567 (19.3)
Obesity^c^	133 (1.6)	74 (4.8)	144 (5.6)	179 (6.1)
Morbid obesity^d^	17 (0.2)	11 (0.7)	46 (1.8)	52 (1.8)
**AHA Severe obesity**				
Class 2^e^	24 (0.3)	16 (1.0)	49 (1.9)	58 (2.0)
Class 3^f^	5 (0.1)	2 (0.1)	10 (0.4)	16 (0.5)

AHF85, Australian Health and Fitness Survey 1985; NNS95, National Nutrition Survey 1995 NCNPA07, Australian National Children’s Nutrition and Physical Activity Survey 2007; NHS12, National Health Survey 2012; IOTF, International Obesity Taskforce; AHA American Heart Association.

^a^Equivalent to age and sex adjusted BMI <25 at 18 years

^b^Equivalent to age and sex adjusted BMI ≥ 25 & <30 at 18 years

^c^Equivalent to age and sex adjusted BMI ≥ 30 & <35 at 18 years

^d^Equivalent to age and sex adjusted BMI ≥ 35 at 18 years

^e^BMI ≥120% and <140% 95th centile or BMI ≥35 and <40

^f^BMI ≥140% 95th centile or BMI ≥40

## Discussion

This study is the first to describe the temporal trends over 27 years in morbid and severe obesity in Australian children. The prevalence of children with morbid/severe obesity increased between 1985 and 2012, but of great public health concern is the proportional increase in morbid/severe obesity among children with obesity. In 1985 morbid obesity represented 11.3% of children with obesity and 22.5% in 2012. A similar trend was seen in children with combined class 2 and 3 severe obesity.

An increased prevalence of children with morbid obesity has been observed elsewhere, with higher rates reported among certain population groups. In 2013 in New Zealand, 2.5% of children had morbid obesity, based upon the IOTF definition, however the prevalence was higher among Pacific Islander (9%) and Maori (5%) children and those living in areas of high deprivation (6%) [11]. In the UK the prevalence of morbid obesity (defined as ≥99.6th centile of the UK 1990 growth charts) from 2006 to 2013 was 4.1% in 4–5 year olds and 5.9% in 10–11 year olds, and higher among children from lower socioeconomic areas, children from black ethnic groups and among older boys [12]. Similarly, in 2011to 2012 in the US, 8% of 2–19 years olds were severely obese, with the prevalence being higher among adolescents and non-Hispanic black males [4].

A plateauing of the proportion of children with overweight or obesity as measured by BMI has been previously reported in Australia [13]. In contrast to the US [4] and Italy [14] our results show a similar trend for morbid/severe obesity. Potentially, the plateau in morbid obesity may reflect the plateau in obesity, that is, efforts to stabilise obesity have slowed the incidence of obese children becoming morbidly obese. Since the early 2000s child obesity prevention has been a public health priority and there has been significant government investment in the early childhood and the primary school sectors [15–18]. While the study findings are positive, a major concern is accessing morbidly obese children through population surveillance surveys. The response rates of the surveys decreased over time, raising a strong possibility of systematic non-response bias. Further, bias may have occurred due to the fact that in the 2012 National Health Survey ~18% of children living in households that agreed to participate in the survey refused to have their height and weight measured. A higher proportion (20.3%) of girls aged 12 to 15 years refused to be measured compared to boys aged 7 to 11 years (16.7%). The implications are not clear, but given the stigma associated with obesity we speculate that that the proportion of children with morbid/severe obesity may be underestimated and the underestimation may differ by sex and age. A further limitation of the study is that the absolute numbers of children with morbid and/or severe obesity are low. For example, for severe obesity class 3 the numbers ranged from two children in 1995 to 16 children in 2012 and hence the magnitude of change should be interpreted with caution. The strength of the study is that it based on four national Australian cross-sectional surveys of measured height and weight.

In this study we applied the IOTF BMI cut-points for morbid obesity [2] and the American Heart Association definition, based on the CDC 2000 growth charts, for severe obesity class 2 and class 3 [3]. In Australia both IOTF criteria and CDC 2000 growth chart cut-points are used in clinical practice and research to identify overweight and obesity. Both definitions indicated a similar trend in the severity of obesity, that is a significant increase between 1995 and 2007, although the proportion of severe obesity at all time periods was approximately 10% higher compared to morbid obesity. These results stress the importance of using the same definition when comparing population data. Nevertheless, both definitions use BMI criteria and selection of cut-points is based on statistical considerations rather than a clear relation with health risk or degree of body fatness [19]. Studies using skinfold and waist circumference measurements suggest that BMI underestimates change in fatness in children [20]. Further research efforts are need to address the relation between BMI and body fat in children.

Based on the NHS 2012 data morbid obesity affects over 30,000 Australian children age 7-15-years. Of concern is that current paediatric obesity services are under-resourced and funding for primary prevention of child obesity is limited [21]. In contrast to overweight and obese children, children with morbid obesity have a worse adverse cardiometabolic risk factor profile, demonstrate early signs of vascular dysfunction and subclinical atherosclerosis and required tertiary intervention. There are currently not enough tertiary paediatric obesity services to respond to the high number of children with morbid obesity, and better paediatrician training in obesity management is needed. The protracted nature of morbid obesity requires policy decisions to invest in coordinated models of care for health-service delivery for the management of morbid obesity in children. In the absence of such policy decisions, Australia is in a precarious situation as greater pressure is placed on limited health care services and managing associated health costs.
